# Association of a nicotinic receptor gene polymorphism with spontaneous eyeblink rates

**DOI:** 10.1038/srep08658

**Published:** 2015-03-02

**Authors:** Tamami Nakano, Chiho Kuriyama, Toshiyuki Himichi, Michio Nomura

**Affiliations:** 1 Graduate School of Frontiers Biosciences, Osaka University, Osaka, Japan; 2 Graduate School of Education, Kyoto University, Kyoto, Japan; 3 Japan Society for the Promotion of Sciences, Tokyo, Japan

## Abstract

Spontaneous eyeblink rates greatly vary among individuals from several blinks to a few dozen blinks per minute. Because dopamine agonists immediately increase the blink rate, individual differences in blink rate are used as a behavioral index of central dopamine functioning. However, an association of the blink rate with polymorphisms in dopamine-related genes has yet not been found. In this study, we demonstrated that a genetic variation of the nicotinic acetylcholine receptor *CHRNA4* (rs1044396) increased the blink rate while watching a video. A receiver operating characteristic analysis revealed that the blink rate predicts a genetic variation in the nicotinic receptor gene with a significant discrimination level (0.66, p < 0.004). The present study suggests that differences in sensitivity to acetylcholine because of the genetic variation of the nicotinic receptor are associated with individual differences in spontaneous eye blink rate.

Spontaneous eyeblink rates greatly vary among individuals from several blinks to a few dozen blinks per minute. A series of studies demonstrated a possible relationship between blink rates and central dopamine activity^1^. First, several dopamine agonists immediately increased blink rates in monkeys and humans^2^,^3^. Second, patients with Parkinson disease resulting from the death of midbrain dopamine neurons had a strikingly low blink rate^1^,^4^. Third, the blink rate correlated with dopamine levels in the caudate nucleus of monkeys^5^. Therefore, the spontaneous eyeblink rate is used as a behavioral index of central dopamine functioning^6^,^7^. This close relationship raises the possibility that dopamine-related genetic variation produces individual differences in blink rates. However, a previous study reported that genetic polymorphisms of dopamine metabolic enzymes and dopamine receptors have no association with blink rates^7^. This raises another possibility that the association between blink rates and dopamine is a secondary phenomenon caused by other factors.

Another important factor influencing the blink rate is nicotine, which was evidenced by the fact that smoking immediately increases the blink rate^1^. The nicotinic receptors, located in the presynapse of the midbrain dopamine neurons, induce dopamine release^8^,^9^, often leading to nicotine addiction. Further, a human genetic study showed that the allelic variation of CHRNA4 (rs1044396) was associated with nicotine addiction^10^. Given that previous studies have revealed that the intake of nicotine from smoking or nicotine gum modulates reflexive blinks through the cholinergic-dopaminergic system^11^,^12^, blink generation might occur through the direct action of nicotine on nicotinic receptors in the brainstem. Therefore, we speculate that a genetic polymorphism of the nicotinic receptor produces the individual differences in spontaneous eyeblink rates. Among several kinds of nicotinic receptor, the α4β2 subunit, the most popular neural nicotinic receptor, has the highest sensitivity to acetylcholine^13^. Previous studies using knockout mice revealed an influence of CHRNA4 gene, which encodes this α4 subunit, on the dopamine function^8^,^14^. Moreover, this CHRNA4 rs1044396 and the dopamine D2 receptor gene, DRD2, interacted with the gray matter volume in the human striatum^15^. These observations suggest that CHRNA4 rs1044396 has an influence on the dopamine function in humans. We accordingly hypothesized that the allelic variation in CHRAN4 (rs1044396) changes the dopaminergic function, resulting in individual differences in the blink rate. To address this hypothesis, the present study examined the relationship between spontaneous eye blink rate and a single-nucleotide polymorphism (SNP) (rs1044396) in the nicotinic receptor CHRNA4.

## Methods

### (a) Participants

One-hundred-four Asian healthy young adults with no smoking habit participated in the present study (53 males and 51 females, aged 20–31 years). One participant was excluded from the analysis because her mean blink rate (61.3 blinks per min) fell outside the range of 3 standard deviations around the mean (19.4 ± 10.1). The study was approved by the review boards of Osaka University and Kyoto University and carried out in accordance with the approved guidelines. All participants provided written informed consent before participation.

### (b) Blink rate analysis

The blinking behavior of the participants was measured by vertical electrooculograms (EOGs) while they freely watched an 8-min video taken from the British television comedy “*The Best Bits*” in “*Rowan Atkinson in Mr. Bean 1*.” This video was used in a previous blink study^16^. Subsequently, we measured the blinking behavior in the resting state for 3 minutes. EOGs were recorded using Ag/AgCl electrodes attached to the left supra- and infraorbital sites. The reference electrode was placed on the left ear lobe. The EOG signals were amplified by a bioelectric amplifier (MP150, BIOPAC Systems, USA) with an AC time constant of 0.3 s, digitized online at a rate of 1000 Hz, and stored on a hard disk. After the experiment, each eye blink was automatically detected according to criteria that were characterized by a combination of a rapid increase of the EOG signal followed by a decrease within 400 ms (see Supplementary Figure 1). Because the present study did not show any sudden stimulus eliciting a reflexive response or request to generate eyeblinks, we regarded the eyeblinks generated by participants while viewing the movie as spontaneous^17^.

The distribution of the inter-blink time interval was calculated for each participant with a bin width of 1 s. The frequency was normalized by the total number of eye blinks. We then compared the distributions of the inter-blink time intervals between the genotypes (CC vs. CT/TT).

The participants were informed in advance that their eye movements would be measured while watching the video stimulus and that they had to answer four multiple choice questions regarding the content of the video at a later time. They were not informed that their blinking was being measured.

### (c) Genotyping

The nails of the participants were collected for genotyping. Genomic DNA extraction was performed using the ISOHAIR DNA extraction kit (NIPPON GENE CO., LTD, Toyama, Japan). Genotyping of *CHRNA4* (rs1044396) was performed by the TaqMan method using a 7500 Real-time PCR System (Applied Biosystems, Foster City, CA, USA). The PCR mixture consisted with 10 μl of TaqMan Genotyping Master Mix (Applied Biosystems), 0.5 μl of assay mix (Applied Biosystems), 40 ng of genomic DNA, and 7.5 μl of water (total volume, 20 μl). PCR was performed using 96-well plates, and the PCR conditions were 50°C for 2 min, 95°C for 10 min, followed by 50 cycles of 92°C for 15 s, and 58°C for 1 min. PCR reactions used two primers, forward (AAAGCCAGGTCCCTCAGCGTC) and reverse (AAGCGAAGCAGCCTGAGGCCT).

## Results

**Figure 1:**
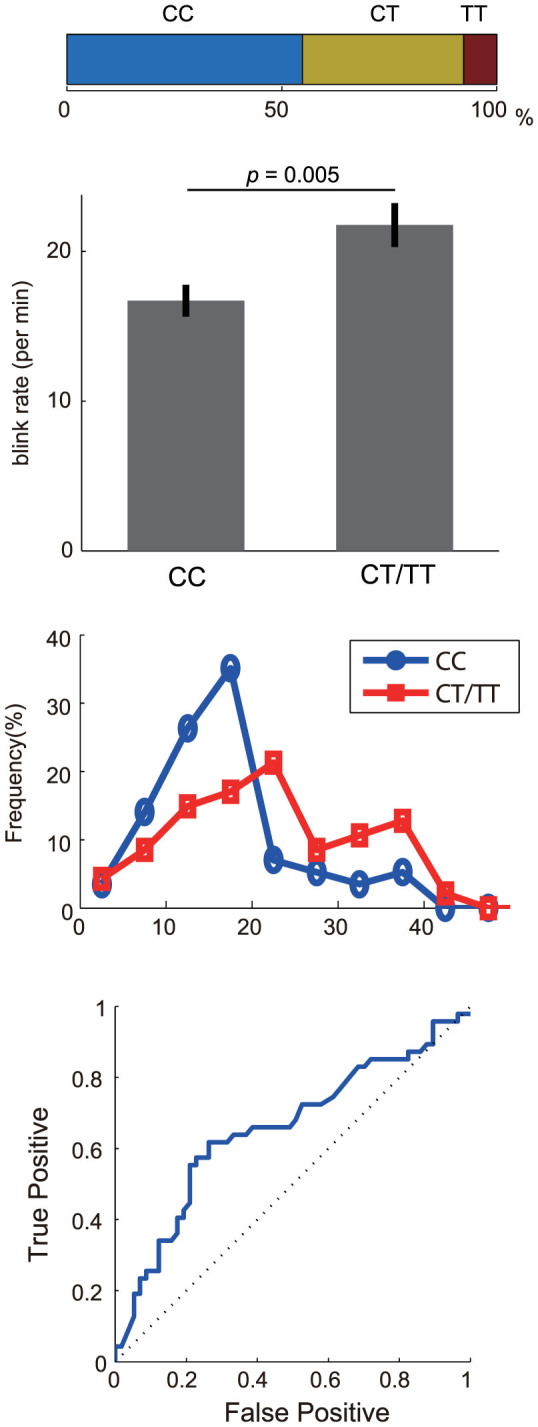
Effects of allelic variation in the nicotinic receptor gene on spontaneous eye blink rates (A) Relative single nucleotide polymorphism frequency of the *CHRNA4* rs1044396 genotype in the present study. (B) Comparison of mean blink rate between the genotype groups. The error bar represents the standard error. (C) Distributions of spontaneous eye blink rates for the CC (blue line) and CT/TT genotypes (red line). (D) Receiver operating characteristic curve for the spontaneous eye blink rate to predict the *CHRNA4* rs1044396 genotype (blue line). For each participant, sensitivity (true positive rate, Y-axis) is plotted against 1 − specificity (false positive rate, X-axis).

The mean blink rate during the rest session was significantly higher than that during the video session (rest, 23.2 blinks/min; video, 19.4 blinks/min; paired t-test, t = 4.0, p < 0.0002), suggesting participants attentively watched the video. The participants were divided into three groups on the basis of the number of C alleles in the *CHRNA4* (rs1044396), two (CC genotype, n = 57; male, n = 32; female, n = 25), one (CT genotype, n = 39; male, n = 17; female, n = 22), and none (TT genotype, n = 8; male, n = 4; female, n = 4), as shown in Figure 1A. This *CHRNA4* SNP frequency is consistent with the Hardy–Weinberg equilibrium (*p* = 0.8). Because the frequency of the TT genotype was low in Asian samples, we divided the enrolled participants into the following two groups: CC and CT/TT. The proportion of CC carriers in males was slightly higher than that in females (males, 0.6; females, 0.5); however, Fisher's exact test showed no significant difference between them (*p* = 0.08). The mean blink rate in males was slightly lower than that in females (males, mean ± S.D., 17.3 ± 8.3; females, 20.7 ± 9.9); however, it did not reach a significant level (two-sample *t* test, *p* = 0.06). The mean accuracy of the questionnaires regarding the video stimuli was almost the same between the two groups (CC genotype, mean ± SD, 81 ± 19%; CT/TT genotype, 81 ± 17%).

The mean blink rate during the video session for the CT/TT genotype (21.7 ± 1.0, mean ± SE) was significantly higher than that for the CC genotype (16.7 ± 0.8, mean ± SE) according to a two-sample *t*-test (t = 2.9, *p* = 0.005, Figure 1B). The distribution patterns of the blink rates for the CC and CT/TT genotypes were distinctive different (Figure 1C). Especially, 80% of CC genotypes was concentrated under the 20 blinks per minute, whereas the CT/TT genotypes dispersedly distributed. A receiver operating characteristic analysis revealed that the blink rate predicts the retention of the T allele with a high discrimination level of 0.66 (bootstrap test, z = 2.9, p < 0.004, Figure 1D). The optimal cutoff point was 17.3 blinks per minute, with 65% of participants with the CC genotype having a blink rate lower than this cutoff, whereas 64% of participants with a CT/TT genotype had a rate exceeding this cutoff. In contrast, the mean blink rate in the resting state for the CT/TT genotype (mean ± SE, 24.1 ± 1.3) was not significantly different from that for the CC genotype (mean ± SE, 22.2 ± 0.9) according to the 2-sample t-test (t = 0.9; p = 0.4).

To evaluate the interaction between gender and genotype, we performed a two-way analysis of variance (ANOVA). We again observed a significant main effect of the genotypes (CC vs. CT/TT, F_1,100_ = 7.2, *p* = 0.009); however, no significant main effect of gender (F_1,100_ = 3.2, *p* = 0.08) and no significant interaction (F_1,100_ = 1.7, *p* = 0.3).

To compare the physical characteristics of blink generation between the genotypes, we analyzed the distribution of the inter-blink time intervals while watching the movie. The distributions in both the genotypes were found to follow Poisson distributions (see Supplementary Figure 2), and no significant difference was observed between the genotypes by ANOVA (*p* = 0.8).

## Discussion

The present study found that the individual differences in the spontaneous blink rate are associated with a SNP in the nicotinic receptor gene *CHRNA4*. The nicotinic receptor is the major presynaptic receptor of the midbrain dopamine neurons, and it induces dopamine release^8^,^9^. A genetic variation in *CHRNA4* enhanced the affinity of the nicotinic receptor for acetylcholine^18^. This raises the possibility that the striatum dopamine level is relatively high in people who carry the CT/TT allele of the *CHRNA4* gene. Considering that the spontaneous blink rate is positively correlated with the striatum dopamine level^5^, we suggest that an increased sensitivity of the nicotinic receptor because of a genetic variation in the *CHRNA4* gene promotes dopamine release in the midbrain, resulting in an increased spontaneous blink rate. Therefore, the association between blink rates and dopamine may in fact be a secondary phenomenon, resulting from nicotinic receptor functioning.

The cholinergic neurons, which are widely distributed in the cortex and thalamus, are involved in the regulation of arousal and attention levels^19^. The previous study reported that genetic variation at rs1044396 improved the performance in multiple tracking and visual searching tasks^20^,^21^. Considering that an increased nicotine level speeds the detection of targets outside the focus of attention, the nicotinic receptor is particularly involved in the process of attentional disengagement from the current target^20^. In line with these previous findings, the present results of reduced blink rate in CC carriers suggest that their attention to the current target is heightened. These observations are mutually consistent, given that eye blinks are actively involved in the process of attentional disengagement during cognitive behavior by momentarily deactivating the visual attention network^16^. Therefore, the spontaneous blink rate may reflect an attentional flexibility mediated by the nicotinic cholinergic system.

Another important finding in the present study is that the distribution of the blink rate in CT/TT carriers is wide, whereas that in CC carriers is sharply centered at 20 blinks per minute. These results suggest that some other factors determine the blink rate in CT/TT carriers. Further investigation is expected to identify gene–gene interactions of CHRNA4 (rs1044396) with other polymorphisms that modulate the blink rate.

The nicotinic acetylcholine system is closely related to various neural diseases, such as Alzheimer disease^22^, Parkinson disease^4^, and nicotine addiction^10^. In particular, several SNP studies with large sample sizes found that the rs1044396 SNP in *CHRNA4* decreases the susceptibility to nicotine addiction^10^. The present study demonstrated that spontaneous blink rate is associated with genetic variation in the neural nicotinic receptor. Because blinking is an obvious and easily measurable behavior, the spontaneous eyeblink rate can provide useful information for predicting nicotinic receptor genotype, which may be necessary for assessing the risk of these neural disease.

## Author Contributions

T.N. and M.N. designed the research. T.N., C.K. and T.H. performed the experiments and analyzed the data. T.N. wrote the main manuscript and created the figure.

## Supplementary Material

Supplementary Information Supplementary Figures
