# Supplementary material for: Association of a nicotinic receptor gene polymorphism with spontaneous eyeblink rates
Source: Sci Rep. 2015 Mar 2;5:8658. doi: 10.1038/srep08658 (PMC4345315; doi:10.1038/srep08658)

Association of a nicotinic receptor gene polymorphism with spontaneous eyeblink rates

Tamami Nakano, Chiho Kuriyama, Toshiyuki Himichi, Michio Nomura

Supplementary Figure1

The eyelid movement in EOG recordings. (A) An original EOG recording data related to blinking behavior from one typical participant. The red circles represent the onset time of eyeblink. (B) An averaged wave form of EOG data for the spontaneous eyeblinks from one typical participant.

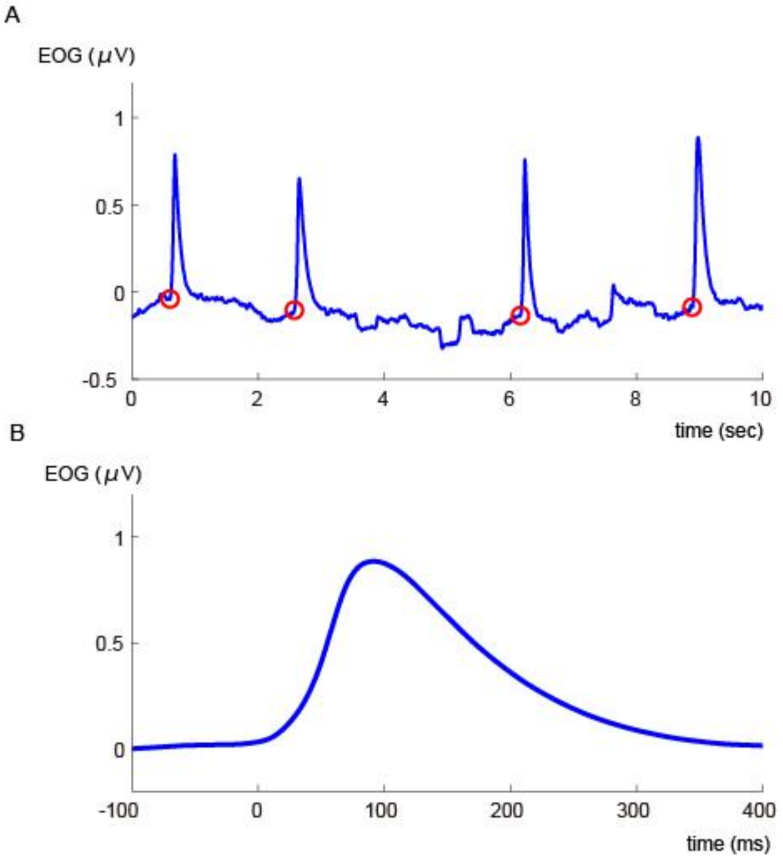

Supplementary Figure 2

The distribution of the inter-blink time intervals in the CC carriers (blue line) and the CT/TT carriers (red line). The error bar represents a standard error. The blink frequency is normalized by the number of blinks for each participant. The two-way ANOVA with factors of the SNP type (CC vs. CT/TT) and time revealed no significant difference between the two genetic groups ($p = 0.8$).

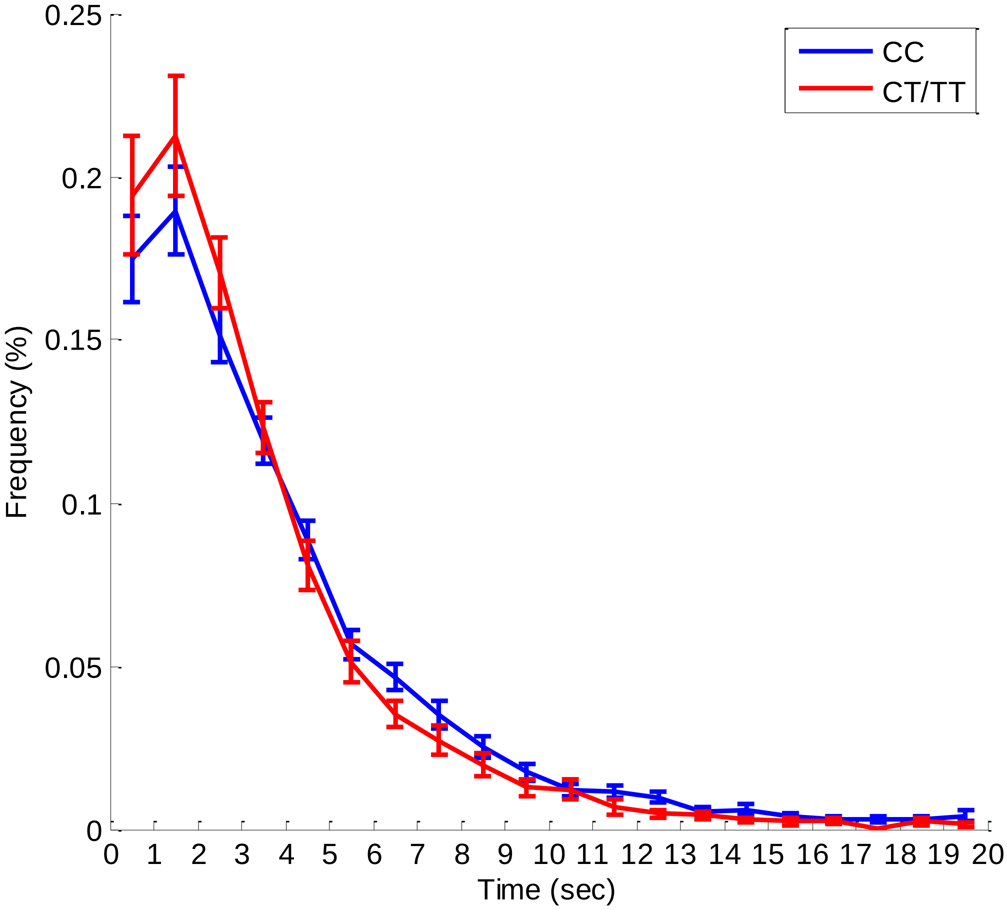

Supplement: Supplementary Information — Supplementary Figures [file srep08658-s1.pdf]
